# The Mechanism of Simvastatin-Mediated M1 Macrophage Polarization Contributing to Osteogenesis and Angiogenesis

**DOI:** 10.3390/biomedicines13061454

**Published:** 2025-06-12

**Authors:** Siyu Zhu, Yunmeng Tong, Jiaqian Huang, Yuzhu He, Wenqi Fu, Yaran Zang, Huiying Liu

**Affiliations:** 1 School of Stomatology, Dalian Medical University, No. 9 West Section, Lvshun South Road, Dalian 116044, China; zhusiyu1994official@hotmail.com (S.Z.); 17860753107@163.com (Y.T.); hjqian922@126.com (J.H.); yuzhuh0723@dmu.edu.cn (Y.H.);; 2 Dalian Key Laboratory of Immune and Oral Development & Regeneration, Dalian Medical University, Dalian 116044, China

**Keywords:** simvastatin, osteogenesis, angiogenesis, macrophage polarization, bone immune

## Abstract

**Background:** The immune response is essential for bone regeneration, and macrophages in the immune microenvironment contribute to bone metabolism and angiogenesis. Emerging evidence demonstrates that simvastatin is a promising candidate for bone repair and promotes bone formation both in vitro and in vivo. However, the effect of simvastatin on macrophages and the following outcomes are still unclear. **Objectives:** This study aimed to investigate the potential immunomodulatory effect of simvastatin on M1 macrophages and its subsequent impact on osteogenesis and angiogenesis. **Methods:** Cell viability was assessed by CCK-8. Osteogenic and angiogenic markers were evaluated by RT-qPCR, Western blotting, and immunofluorescence. M1 macrophage phenotype was analyzed by flow cytometry. Osteogenesis was examined by histological staining, and angiogenic capacity was assessed using functional assays. **Results:** The present study found that simvastatin decreased M1 macrophage markers (CD86) and stimulated M1 macrophages to express high levels of pro-regenerative cytokines (BMP-2 and VEGF). In addition, simvastatin promoted osteogenic differentiation in MC3T3-E1 cells and angiogenic gene expression in HUVECs. Importantly, simvastatin enhanced the osteogenic capacity of MC3T3-E1 and the angiogenic potential of HUVECs by inhibiting M1 macrophage polarization in vitro. **Conclusions:** We demonstrated that simvastatin could confer favorable bone immunomodulatory properties and influence the crosstalk behavior between immune cells and osteoblasts and vascular endothelial cells to promote bone healing.

## 1. Introduction

When tissue injury, infection, or material implantation occurs, acute and chronic inflammation gradually appears [1]. Long-term inflammation can lead to fibrous tissue wrapping the surface of biomaterials, isolating the external environment, and thus osseointegration and implantation failure [2]. Various inflammatory cells are involved in the process of tissue healing, including T cells, B cells, macrophages, neutrophils, and so on [3]. Among them, macrophages play a central role in coordinating the immune inflammatory response and tissue repair [4]. In addition, macrophages can be polarized into “classically activated” M1 and “alternatively activated” M2, and the effect is regulated by the microenvironment [5,6]. M1 macrophages, induced by interferon (IFN)-γ or lipopolysaccharide (LPS, endotoxin) or tumor necrosis factor (TNF)-α, promote inflammation by producing pro-inflammatory cytokines like interleukin (IL), TNF, and IFN [7,8,9], inducible nitric oxide synthase (iNOS), nitric oxide (NO), and reactive oxygen species (ROS) [10,11]. These signaling cytokines play an important role in processes related to tissue regeneration [12]. Therefore, the host immune response plays a key role in the success of tissue regeneration. 

In the process of bone tissue repair, several reactions are involved, including immunoinflammatory modulation, angiogenesis, osteogenic differentiation, and bone regeneration [13,14]. Bone is a highly vascularized tissue, and studies have shown that a rich network of blood vessels not only provides nutrients and oxygen delivery but also releases paracrine signals that regulate the growth, differentiation, and regeneration of different cell types, such as bone cells, which are conducive to the subsequent formation of new bone [15,16,17]. Histological analyses found that osteoblasts and bone progenitor cells were densely distributed near the neovasculature in the defect area, confirming a close spatial and temporal link between osteogenesis and angiogenesis [18,19]. Evidence suggests that macrophages are the main cells in the immune regulation of bone regeneration [20]. Following bone tissue injury, macrophages infiltrate into the injury area. They are activated as M1-type macrophages that secrete high levels of pro-inflammatory cytokines, inhibit osteoblast activity, and enhance osteoclast differentiation, thereby hindering new bone formation [21]. In addition to secreting cytokines, M1 macrophages are also precursors of osteoclasts, showing the potential to differentiate into osteoclasts, so the increase in M1 macrophages would contribute to bone resorption and osteoclast activity [22,23]. Moreover, literature reports show that M1 macrophages have an inhibitory effect on angiogenesis during bone defect repair [24]. For example, Huang et al. [25] showed that exosomes derived from bone marrow mesenchymal stem cells were beneficial not only for osteogenesis but also for angiogenesis, by modulating M1 macrophages. Thus, this close crosstalk between immune response, angiogenesis, and osteogenesis is a key factor in bone tissue healing.

With the continuous study of osteoimmunology, more and more scholars are trying to discover new mechanisms of old drugs and apply them to the fields of macrophage polarization and bone tissue repair [26,27]. Statins are reductase inhibitors of Hydroxymethyl glutaryl-CoA (HMG-CoA), mainly used in the treatment of hypercholesterolemia and coronary artery disease, with the advantages of safety, effectiveness, and a low price [28,29]. In recent years, the potential applications of simvastatin have been studied, especially the effects on bone formation and angiogenesis [30,31]. Mundy et al. [32] first reported that simvastatin stimulated bone formation in vitro and in rodents. Zhu et al. [33] reported that simvastatin application mobilized endothelial progenitor cells and induced angiogenesis in a diabetic hindlimb ischemia rat model. Previous research reported that statins could affect macrophages, and data from Rebecca Linnenberger et al. [34] suggested that simvastatin had a unique immunomodulatory effect on macrophage polarization. Although the above findings suggest a potential regulatory role for simvastatin on osteogenesis, angiogenesis, and macrophage polarization, whether simvastatin is involved in bone immune regulation during bone healing and angiogenesis in the immune microenvironment remains unknown. This study explains the deep mechanism of simvastatin on macrophage polarization, osteogenesis, and angiogenesis and emphasizes the key role of simvastatin in regulating bone regeneration and angiogenesis through modulating macrophage polarization, providing more support for the pleiotropic effects of the first-line drug simvastatin.

## 2. Materials and Methods

### 2.1. Reagents

Simvastatin and LPS were obtained from Sigma-Aldrich (Burlington, MA, USA). Mouse macrophage cells (RAW 264.7, ATCC® TIB-71™), mouse embryonic osteoblast precursor cells (MC3T3-E1, ATCC® CRL-2593™), and human umbilical vein endothelial cells (HUVECs, ATCC® PCS-100-010™) were purchased from the American Type Culture Collection (Manassas, VA, USA). α-Minimum Eagle‘s Medium (α-MEM), Dulbecco‘s Modified Eagle Medium (DMEM), Endothelial Cell Medium (ECM), Fetal Bovine Serum (FBS), and Phosphate Buffered Saline (PBS) were purchased from Gibco (Tampa, FL, USA). Pancreatin and Penicillin–Streptomycin (PS) were obtained from Hyclone (Logan, UT, USA). The CCK8 assay kit and Alkaline Phosphatase (ALP) Assay Kit was obtained from Beyotime (Shanghai, China). The Transcriptor First Strand cDNA Synthesis Kit and SYBR Green Master Mix were purchased from Takara (Kusatsu, Japan). The primary antibodies for Glyceraldehyde-3-Phosphate Dehydrogenase (GAPDH), Bone Morphogenic Protein-2 (BMP2), and Vascular Endothelial Growth Factor (VEGF) were purchased from Santa Cruz Biotechnology (San Jose, CA, USA). The primary antibodies for CD86, CD31, Hypoxia-Inducible Factor-1α (HIF-1α), and the secondary antibodies (Horseradish Peroxidase-Conjugated Anti-Rabbit Immunoglobulin G) were obtained from Abcam (Cambridge, UK). The enhanced chemiluminescence reagent (ECL) was purchased from Thermo Fisher Scientific (Waltham, MA, USA). The DAPI (4′,6-Diamidino-2-Phenylindole) Staining Kit was obtained from Abbkine Scientific Co., Ltd. (Atlanta, GA, USA). The Matrigel Matrix was obtained from Corning Inc. (New York, NY, USA). TRIzol reagent, Alexa Fluor® 488-conjugated goat anti-rabbit IgG and Alexa Fluor® 594-conjugated goat anti-mouse IgG secondary antibodies were purchased from Invitrogen (Carlsbad, CA, USA), and the fluorescent quantitative PCR kit (SYBR® Premix Ex Taq™ II) was obtained from Takara Bio Inc. (Kusatsu, Japan). Primary antibodies against GAPDH and BMP-2 were purchased from Santa Cruz Biotechnology (Dallas, TX, USA), while antibodies against CD86 and VEGF were obtained from Abcam (Cambridge, UK).

### 2.2. Cell Culture

MC3T3-E1 cells were cultured in α-MEM supplemented with 10% FBS and 1% PS. RAW264.7 cells were maintained in a DMEM enriched with 10% FBS. Meanwhile, HUVECs were grown in an ECM fortified with 5% FBS, 1% PS, and 1% Endothelial Cell Growth Supplement (ECGS). These three cell types were incubated in an environment containing 5% CO2 at 37 °C. Cells were passaged once they reached 80% confluence and were utilized between the 10th and 13th generations.

### 2.3. Proliferation Assay

The proliferation test of RAW264.7 cells, MC3T3-E1 cells, and HUVECs cultured in a medium with various concentrations of simvastatin solution (0 μM, 0.1 μM, 0.5 μM, 1 μM, 2 μM) was performed using a CCK8 assay. For the proliferation assay, the cells were incubated for 1, 2, and 3 days. After each incubation period, 90 μl of fresh culture medium was mixed with 10 μl of CCK8 reagent and added to cells. The cells were then incubated for an h. Finally, the optical density at 450 nm was measured and recorded using a microplate reader (BioTek Synergy H1, Temecula, CA, USA).

### 2.4. Flow Cytometry of Simvastatin-Treated M1 Type Macrophage

First, 1 μg/ml LPS was used for 24 h to polarize the macrophages towards the M1 type. M1-type macrophages were stimulated with different concentrations of simvastatin (0 μM, 0.5 μM, 1 μM, 2 μM) for the detection of CD86 expression. Following the treatment, cells were fixed with 4% paraformaldehyde for 15 min. Subsequently, the primary antibody CD86 was added, and the cells were incubated at 4 °C for 60 min. The secondary antibody labeled with fluorescent dye 488 was then applied, and the cells were resuspended and incubated at 4 °C for another 60 min in the dark. Afterward, the cells were analyzed using a flow cytometer, and the resulting data were processed using FlowJo software (version 10.8.1, FlowJo LLC, Ashland, OR, USA).

### 2.5. Western Blot Analysis of Simvastatin-Treated M1 Type Macrophage

M1-type macrophages were stimulated with different concentrations of simvastatin (0 μM, 0.5 μM, 1 μM, 2 μM) for 24 h to detect the expression of BMP-2 by Western blot, and proteins were extracted using cell lysate RIPA (Radio-Immunoprecipitation Assay) containing 1% protein phosphatase inhibitor and 1% protease inhibitor. Protein concentration was determined using a BCA Protein Assay Kit, and a loading buffer was added to denature the proteins. Equal amounts of protein were separated by SDS-PAGE and transferred to a polyvinylidene fluoride membrane (PVDF membrane). The PVDF membrane was blocked with 5% milk for 1 h. Subsequently, the primary antibodies and the membrane were incubated overnight at 4 °C, followed by incubation with the corresponding secondary antibodies for 1 h. Finally, the ECL was applied. The primary antibodies utilized in this study were anti-BMP-2 (1:200) and anti-GAPDH (1:500).

### 2.6. Co-Culture Method

MC3T3-E1 cells or HUVECs were seeded in 12-well plates at a density of 1 × 105 cells per well, while simvastatin-treated M1-type macrophages were seeded in the upper chamber of a 12-well transwell insert (0.4 μm pore size) at a density of 5 × 104 cells per well. The transwell chamber containing RAW264.7 cells was then placed into the 12-well plate with MC3T3-E1 cells or HUVECs for co-culture.

### 2.7. ALP Activity and Alizarin Red Staining Assay of MC3T3-E1

For the assessment of the influence of simvastatin on MC3T3-E1, including ALP activity and calcium deposition, cells were treated with simvastatin solution at concentrations of 0 μM, 0.5 μM, 1 μM, and 2 μM for 3 days and then replaced with a drug-free osteogenic induction medium for 7 days and 14 days. After 7 days of osteogenic induction, cells were washed twice with PBS and lysed with the lysis buffer provided in the kit. The cell lysates were then incubated with the p-nitrophenyl phosphate (pNPP) substrate solution at 37 °C for 30 min. The reaction was terminated, and absorbance was measured at 405 nm using a microplate reader. The total protein content in each sample was quantified using the BCA Protein Assay Kit (Beyotime, Shanghai, China). The relative ALP activity was calculated by normalizing the absorbance value (OD405) to the corresponding protein concentration (U/mg protein).

Following 14 days of osteogenic induction culture, alizarin red staining was employed to assess calcification deposition in each group, cells were fixed with a 4% formaldehyde solution for 15 min and subsequently stained with a 2% alizarin red staining solution, stained images were captured using an inverted optical microscope. Thereafter, a 10% cetylpyridinium chloride solution was added to elute the bound alizarin red staining solution, and the optical density (OD) value of alizarin red was measured using a microplate reader at a wavelength of 562 nm [35].

For the assessment of the influence of simvastatin-induced immune microenvironment on MC3T3-E1 calcium deposition, cells were co-cultured with simvastatin-treated M1-type macrophages for 7 days, followed by the replacement of the medium with osteogenic induction medium to culture the MC3T3-E1 cells for an additional 14 days, and the samples were then used for the detection of alizarin red staining. 

### 2.8. Migration Assay of HUVECs

To assess the impact of varying concentrations of simvastatin on the migration capacity of HUVECs, cells were seeded in 12-well plates and cultured until the cells converged the plates. The cells were aligned vertically along the “crossline” with a sterile pipette tip, the scratched cells were washed, then the HUVECs were subsequently cultured in a solution containing simvastatin for 24 h, and images were taken 24 h after cultivation under a microscope. 

To assess the impact of simvastatin-induced immune microenvironment on HUVECs migration, the cells were aligned vertically along the “crossline” with a sterile pipette tip, then the HUVECs and simvastatin-treated M1-type macrophages were co-cultured in a transwell insert for 24 h. The cell migration rate was calculated using ImageJ software (version 1.53t, National Institutes of Health, Bethesda, MD, USA).

### 2.9. Tubule Formation Experiments of HUVECs

For the detection of the influence of simvastatin on HUVEC tubule formation, cells were seeded in 12-well plates at a density of 5 × 104 cells per well and treated with varying concentrations of simvastatin (0 μM, 0.1 μM, 0.5 μM, 1 μM, 2 μM) for 12 h. After that, the cells were reseeded on Matrigel at a density of 2×104 cells per well and incubated for 6 h. Image J software (version 1.53t, National Institutes of Health, Bethesda, MD, USA) was then utilized to analyze the number of tubular nodes, tubular branches, and the total length of tubular branches.

To detect the influence of the simvastatin-induced immune microenvironment on HUVEC tubule formation, simvastatin-treated M1-type macrophages were co-cultured with HUVECs on Matrigel for 6 h, and the HUVECs were photographed.

### 2.10. Real-Time qPCR

To evaluate the effects of simvastatin on macrophage polarization, osteogenesis, and angiogenesis-related gene expression, total RNA was extracted from simvastatin-treated M1-type macrophages by the Trizol method (AG, China), reverse transcription was performed from 1000 ng of total RNA to cDNA using reverse transcription kit (AG, China), and quantitative RT-PCR was performed using a fluorescent quantitative PCR kit (AG, China). The primers for the qRT-PCR experiments included GAPDH, BMP-2, CD86, and VEGF (AG, China). GAPDH was used as a normalized reference, and the relative mRNA expression level of the target gene was calculated using the 2^−ΔΔCt^ method. The primer sequences were designed and synthesized in Table 1. 

To evaluate the effects of simvastatin on osteogenesis-related gene expression, MC3T3-E1 cells were treated with a simvastatin solution at concentrations of 0 μM, 0.5 μM, 1 μM, and 2 μM for 3 days, and then replaced with drug-free osteogenic induction medium for 7 days and 14 days. After 7 and 14 days of osteogenic induction culture, mRNA expressions of bone formation-related genes ALP and RUNX-2 were analyzed. The primer sequences are listed in Table 1. 

To evaluate the effects of simvastatin on angiogenesis-related gene expression, HUVECs were seeded in 6-well plates with 1 × 105/well cells. On the second day, different concentrations of simvastatin were added to HUVECs for 3 days to detect the mRNA expression of CD31, VEGF, and HIF-1α. The primer sequences for each transcript are detailed in Table 1. 

To evaluate the effects of simvastatin-induced immune microenvironment osteogenesis-related gene expression, MC3T3-E1 cells were co-cultured with simvastatin-treated M1-type macrophage cells for 5 days, followed by the replacement of the medium with osteogenic induction medium to culture the MC3T3-E1 cells for an additional 7 and 14 days. At each time point, total RNA was extracted to analyze the mRNA expression of bone formation-related genes ALP and RUNX2. 

To evaluate the effects of the influence of the simvastatin-induced immune microenvironment angiogenesis-related gene expression, HUVECs were co-cultured with simvastatin-treated M1-type macrophages for 5 days to detect the mRNA expression of CD31, VEGF, and HIF-1α. 

### 2.11. Immunofluorescence Staining

To investigate the effects of simvastatin on RAW264.7 macrophage polarization, cells were seeded in 48-well plates at a density of 1 × 104 cells per well and treated with different concentrations of simvastatin (0 μM, 0.1 μM, 0.5 μM, 1 μM, 2 μM) for 12 h, and then replaced with culture medium containing 1 μg/ml LPS for 24 h to polarize the macrophages towards M1 type macrophages. Cells were fixed with 4% paraformaldehyde at 37 °C for 20 min and incubated with a blocking solution containing 2.5% BSA and 10% FBS at 37 °C for 60 min, then cells were incubated overnight at 4 °C with primary anti-CD86 (1: 100) and were incubated with the corresponding fluorescein secondary antibody at 37 °C for 60 min, and finally stained with DAPI for 10 min. The cells were observed and photographed under an inverted fluorescence microscope.

To investigate the effects of simvastatin on HUVECs’ angiogenesis, cells were plated in 24-well plates at a cell density of 2 × 104/well. HUVECs were treated with different concentrations of simvastatin for 3 days, and the fluorescence intensity expression of CD31, VEGF, and HIF-1α was detected.

To investigate the effects of simvastatin-induced immune microenvironment on HUVECs’ angiogenesis, cells were co-cultured with simvastatin-treated M1-type macrophages for 3 days of co-culture and photographed under a confocal microscope. 

### 2.12. Statistical Analysis

All statistical analyses were conducted using GraphPad Prism (version 10.0, GraphPad Software, La Jolla, CA, USA) and SPSS Statistics (version 29.0, IBM Corp., NY, USA). To assess differences between two groups, an unpaired Student’s *t*-test was applied. For multiple group comparisons, a one-way ANOVA followed by Tukey’s post hoc test was performed to assess statistical significance. Data are expressed as the mean ± standard deviation (SD) for parametric results, or as 95% confidence intervals for nonparametric outcomes. Statistical significance was set at * *p* < 0.05, ** *p* < 0.01, *** *p* < 0.001, unless otherwise indicated. All experiments were conducted in triplicate and independently repeated at least three times to ensure reproducibility.

## 3. Results

### 3.1. Osteogenesis Effects of Simvastatin on MC3T3E1 Cells

**Figure 1:**
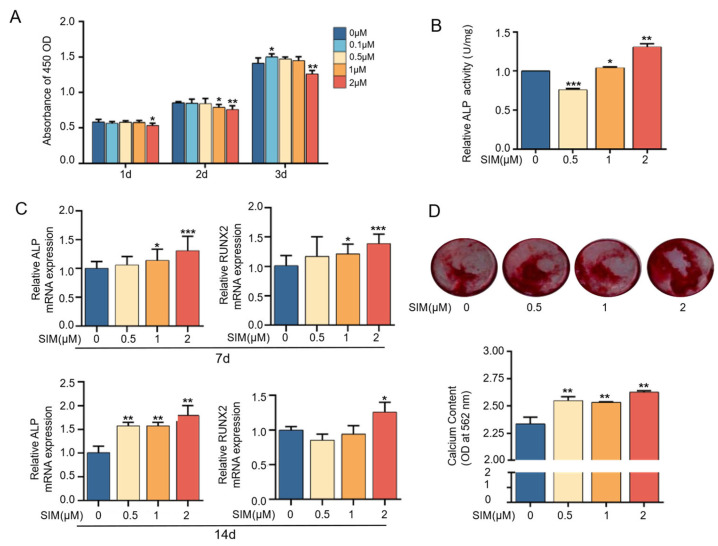
Simvastatin promotes MC3T3-E1 cells’ osteogenic differentiation ability. (**A**) Cell proliferation of MC3T3-E1 treated with simvastatin for 1, 2, and 3 days. (**B**) ALP activity of MC3T3-E1 cells treated with simvastatin for 7 days. (**C**) Expression of osteogenic-related genes of MC3T3-E1 cells treated with simvastatin for 7 and 14 days. (**D**) Alizarin red staining and quantitative analysis of MC3T3-E1 cells treated with simvastatin after 14 days of osteogenesis induction. * *p* < 0.05, ** *p* < 0.01, *** *p* < 0.001. SIM, simvastatin.

A CCK-8 assay was performed to assess the cell proliferation of MC3T3-E1 with different concentrations of simvastatin. As shown in Figure 1A, when increasing the culture time, 0.1 μM of simvastatin showed a significant promoting effect on MC3T3-E1 cell proliferation. Still, as the drug concentration increased, cell proliferation was inhibited, especially at a concentration of 2 μM, suggesting that high concentrations of simvastatin have a certain degree of cytotoxicity. To observe the effect of simvastatin on the osteogenic differentiation of MC3T3-E1 cells, we assessed the effect of simvastatin on ALP activity. Figure 1B showed that simvastatin at 0.5 μM inhibited ALP activity, but as the concentration increased, ALP activity increased and reached a maximum at 2 μM. The results in Figure 1C showed that after simvastatin treatment, the expression of osteogenic differentiation-related factors ALP and Runx2, which represent the osteogenic differentiation ability of the cells, were increased after 7 and 14 days of culture. Compared to the control group, ALP and Runx2 expression increased most when cells were treated with 2 μM of simvastatin, and the difference was significant. Compared with day 7, the expression of ALP increased on day 14, while the change in RUNX2 was not significant. This may be because RUNX2 is an early-stage marker of osteogenic differentiation, while ALP is a mid-stage marker that plays a key role in matrix maturation and mineralization (Figure 1C). Meanwhile, the analysis of cellular bone mineralization ability showed that different concentrations of simvastatin were able to promote mineralization to varying degrees compared to cells without simvastatin treatment; the best performance was achieved when the concentration reached 2 μM, with the highest amount of calcium deposition (Figure 1D). The above results show that simvastatin promoted the osteogenic differentiation of MC3T3-E1 cells, especially at 2 μM, consistent with previous findings [36].

### 3.2. Effects of Different Concentrations of Simvastatin on the Proliferation, Migration, and Tube Formation Ability of HUVEC Cells

**Figure 2:**
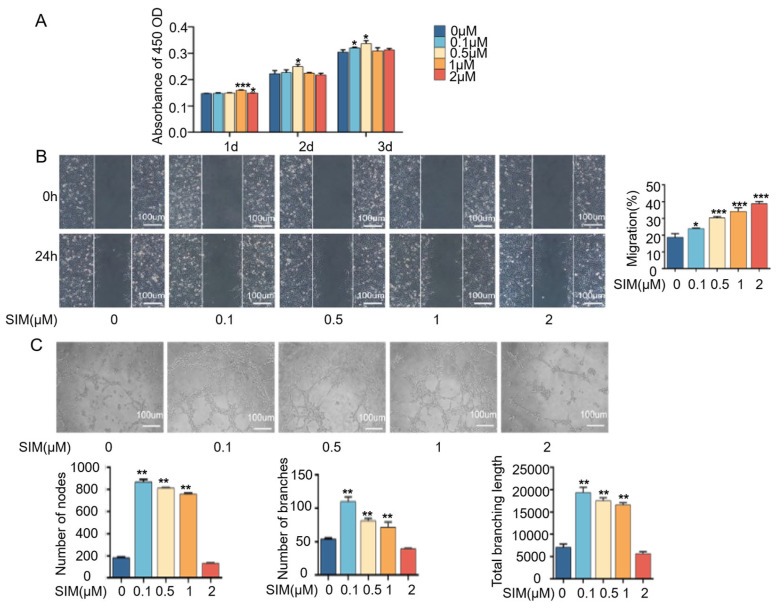
Simvastatin promotes HUVECs’ angiogenic capability. (**A**) Cell proliferation of HUVECs with simvastatin for 12 h, 24 h, 48 h, and 72 h. (**B**) Representative images and relative quantification of wound healing assays of HUVECs with simvastatin treatment (scale of 100 μm). (**C**) Representative images and relative quantification of node number, branch number, and total branching length of tube formation assay (scale of 100 μm). * *p* < 0.05, ** *p* < 0.01, *** *p* < 0.001. SIM, simvastatin.

As shown in Figure 2A, when the concentration was lower than 1 μM, the proliferation of HUVECs increased in a time-dependent manner with the increasing drug concentration. At 24 h, 1 μM of simvastatin significantly promoted cell proliferation compared to the control group, while at 48 and 72 h, 0.5 μM showed the most pronounced effect. However, high doses of simvastatin (both 5 μM and 10 μM) appeared to have a significant inhibitory effect on HUVEC proliferation at 48 and 72 h. 

Next, we tested the angiogenic ability of simvastatin by wound healing and cell tube formation assay. The faster the cell migration rate, the more favorable it is for the healing of injured tissues. Figure 2B shows the qualitative and quantitative results of the migration ability of HUVECs under simvastatin treatment. After 24 h of simvastatin treatment, HUVECs gradually moved toward the cell-free scratched area. Simvastatin could improve the migration ability of HUVECs, and the migration rate was faster with the increase in drug concentration. Figure 2C shows that after treatment with different concentrations of simvastatin, cells in all groups interconnected to form strips, which in turn formed a vascular lattice-like structure. Among them, there were more lattice-like structures in the 0.1 μM, 0.5 μM, and 1 μM groups. Almost all the cells in the 0.1 μM group were interconnected, and the tubule formation was thicker, while in the control group and the 2 μM group, there were still many cells not connected, with fewer lattice-like structures and less obvious tubule formation. A quantitative analysis of the number of tubule nodes, the number of tubule branches, and the total length of tubule branches showed the same results. The above results verified the promotional effect of low concentrations of simvastatin on the vascularization of HUVECs.

### 3.3. Effects of Different Concentrations of Simvastatin on the Secretion of Angiogenesis-Related Factors

**Figure 3:**
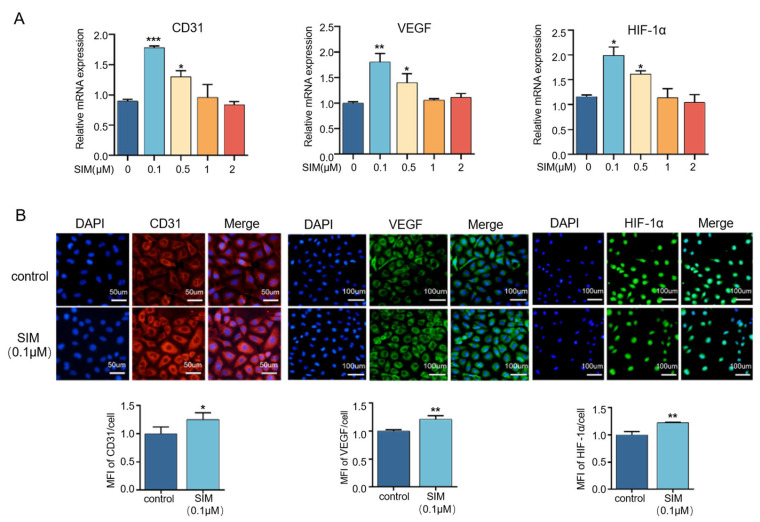
Simvastatin increases the expression of angiogenic-related genes in HUVECs. (**A**) Detection of CD31, VEGF, and HIF-1α expression in HUVECs after 3 days of simvastatin treatment. (**B**) Representative images of immunofluorescence staining of HUVECs treated with simvastatin after 3 days for CD31, VEGF, and HIF-1α and quantification of expression (a scale of 100 μm) * *p* < 0.05, ** *p* < 0.01, *** *p* < 0.001. SIM, simvastatin; MFI, Mean Fluorescence Intensity.

As shown in Figure 3A, a simvastatin concentration of 0.1 μM significantly increased the expression of VEGF, CD31, and HIF-1α compared with the control group, but their expression levels decreased as the drug concentration increased further. To further verify, immunofluorescence staining was also performed to detect the protein expression of angiogenesis-related markers, including VEGF, CD31, and HIF-1α. The fluorescence intensities of intracellular VEGF, CD31, and HIF-1α were increased after treatment with simvastatin at a concentration of 0.1 μM (Figure 3B). These results confirmed the promotional effect of simvastatin on angiogenesis.

### 3.4. Simvastatin Modulates Macrophage Polarization

**Figure 4:**
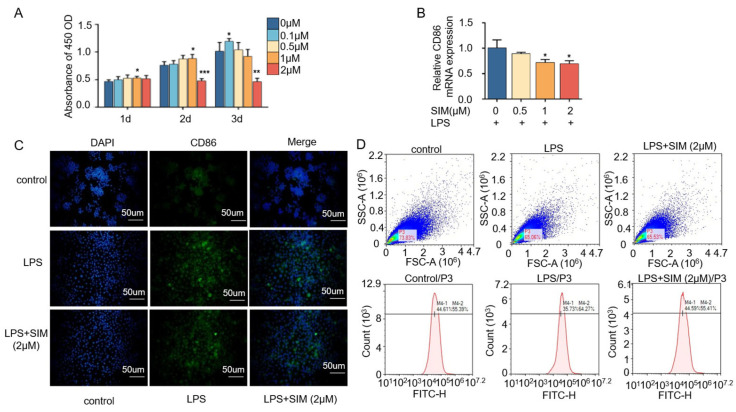
Simvastatin inhibits M1 macrophage polarization in RAW264.7 cells. (**A**) Cell proliferation of RAW264.7 treated with simvastatin for 1, 2, and 3 days. (**B**) Detection of CD86 expression in RAW264.7 cells treated with simvastatin. (**C**) Immunofluorescence staining of CD86 on RAW264.7 cells treated with simvastatin (scale of 50 μm). (**D**) Flow cytometry analysis of the expression of CD86 treated with simvastatin. * *p* < 0.05, ** *p* < 0.01, *** *p* < 0.001. SIM, simvastatin.

The macrophage-mediated immune microenvironment plays a critical role in osteogenesis and angiogenesis. To assess the effect of simvastatin on macrophage polarization, RAW264.7 cells were stimulated with different concentrations of simvastatin. Figure 4A shows that simvastatin promoted cell proliferation as the concentration and time increased, reaching a peak at a concentration of 0.1 on the third day and then decreasing. It is worth noting that a concentration of simvastatin higher than 1 μM had an inhibitory effect on cells. 

Then, we examined the effect of simvastatin on macrophage polarization. Figure 4B shows that simvastatin reduced the expression of M1 macrophage biomarker CD86 in a concentration-dependent manner compared with the control group, implying that the ability of RAW264.7 to differentiate into M1-type macrophages was reduced. As shown in Figure 4C by immunofluorescence staining, the LPS group exhibited an enhanced CD86 signal labeled with green fluorescence compared to the control group, while the fluorescence intensity was decreased in the LPS + SIM (2 μM) group compared with the other two groups. The results of flow cytometry are shown in Figure 4D. The percentage of CD86 positive cells in the control group was 55.39%, and in the LPS group, it was significantly increased to 64.27%, whereas in the simvastatin-treated group, the percentage of CD86 positive cells decreased to 55.41%. These results suggest that simvastatin inhibits macrophage M1 polarization. 

### 3.5. Simvastatin Promotes Osteogenesis by Regulating Macrophage Polarization

**Figure 5:**
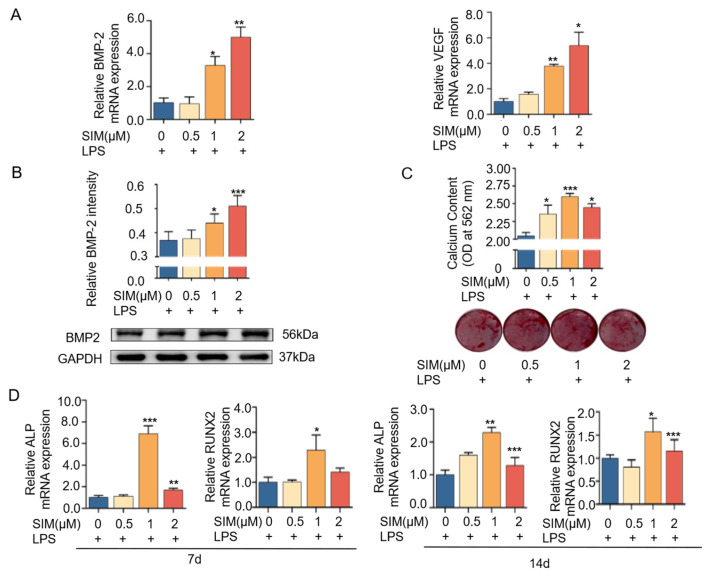
Simvastatin modulates macrophage polarization to promote osteogenesis. (**A**) Detection of BMP2 and VEGF expression in RAW264.7 cells after 12 h of simvastatin treatment. (**B**) Western blotting assay and quantification of BMP2 expression in RAW264.7 cells treated with simvastatin. (**C**) Alizarin red staining and quantitative analysis of co-cultured MC3T3-E1 cells and RAW264.7 pretreated with simvastatin after 14 days of osteogenesis induction. (**D**) Detection of ALP and RUNX2 expression in the co-culture of MC3T3-E1 cells and RAW264.7 pretreated with simvastatin after 7 and 14 days of osteogenesis induction. * *p* < 0.05, ** *p* < 0.01, *** *p* < 0.001. SIM, simvastatin.

Next, we speculated whether the regulatory effect of simvastatin on macrophage polarization affected macrophage secretion of osteogenesis and angiogenesis-related factors. Figure 5A shows a dose-dependent increase in the expression of BMP2 and VEGF compared with the non-simvastatin-treated control group. Western blot results showed that the expression level of BMP2 increased with the increase in simvastatin concentration compared with the control group (Figure 5B). To further evaluate our hypothesis on whether simvastatin could promote osteogenesis by modulating macrophage polarization, we co-cultured macrophages with MC3T3-E1 cells after treatment with different concentrations of simvastatin. Quantitative and qualitative results for detecting mineralization of calcium nodules are shown in Figure 5C; the bone-contributing effect peaked at a concentration of 1 μM, while it declined when the concentration was increased to 2 μM. Figure 5D shows the same trend; after both 7 and 14 days of co-culture, the mRNA expression of ALP and Runx2 increased the most at 1 μM. These results suggest that relatively low concentrations of simvastatin can not only play a role in promoting osteogenic differentiation of preosteoblasts alone but also promote the formation of new bone by regulating macrophage polarization.

### 3.6. Simvastatin Regulates Macrophage Polarization to Promote Angiogenesis

**Figure 6:**
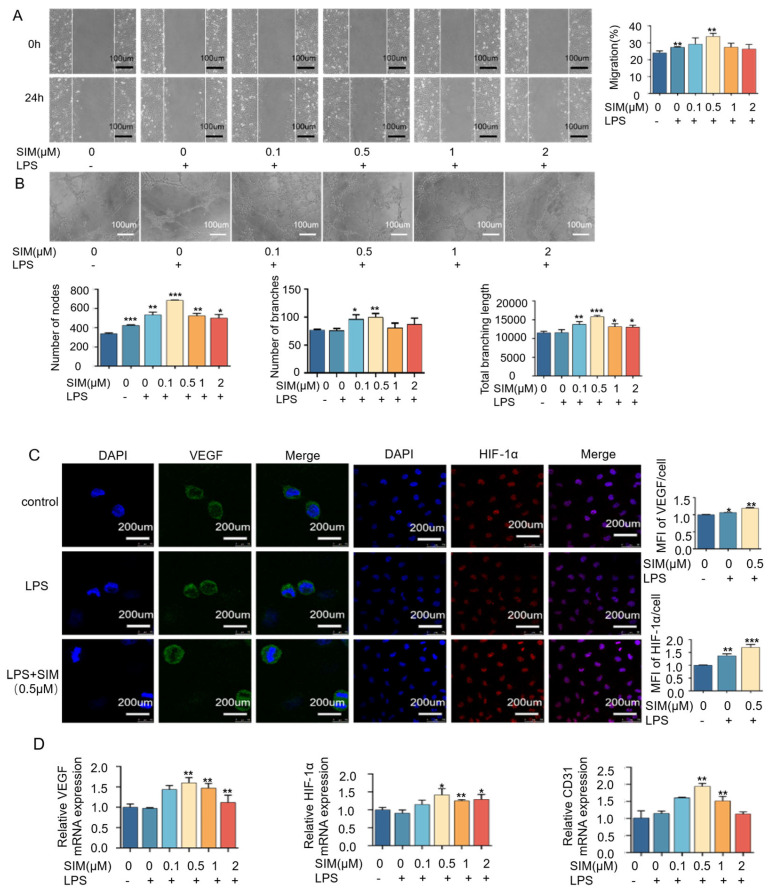
Simvastatin regulates macrophage polarization to promote angiogenesis. (**A**) Representative images and relative quantification of wound healing assays of co-cultured HUVECs and RAW264.7 with a simvastatin treatment (scale of 100 μm). (**B**) Representative images and relative quantification of node number, branch number, and total branching length of tube formation assay of co-cultured HUVECs and RAW264.7 with simvastatin treatment (scale of 100 μm). (**C**) Representative images of immunofluorescence staining of co-cultured HUVECs and RAW264.7 pre-treated with or without 0.5 μM simvastatin after 3 days for VEGF (green) and VEGF (red) and quantification of expression (scale of 200 μm). (**D**) Detection of CD3, VEGF, and HIF-1α expression in co-cultured HUVECs and RAW264.7 pretreated with simvastatin after 5 days. * *p* < 0.05, ** *p* < 0.01, *** *p* < 0.001. SIM, simvastatin.

To test whether simvastatin-mediated macrophage polarization could have a pro-angiogenic effect, simvastatin-treated macrophages were co-cultured with HUVECs. Firstly, the migratory ability of HUVECs was examined, and the 0.1 μM and 0.5 μM simvastatin significantly increased the rate of cell migration but decreased at 1 μM and 2 μM (Figure 6A). Also, 0.1 μM and 0.5 μM of simvastatin showed larger numbers of tubule nodes, tubule branches, and a longer total tubule branch length compared with the control and LPS groups and the rest of the two groups (Figure 6B). Our results demonstrated that low concentrations of simvastatin could increase HUVECs’ migration rate and tube formation capacity by mediating macrophage polarization. Immunofluorescence results showed that both VEGF and HIF-1α exhibited stronger fluorescence intensity at a drug concentration of 0.5 μM compared with the control and LPS groups (Figure 6C). For the detection of the expression level of angiogenesis-related factors, whose results are shown in Figure 6D, the addition of simvastatin could effectively increase the expression level of VEGF, CD31, and HIF-1α in the co-cultured cells. Among them, VEGF, CD31, and HIF-1α were best expressed under 0.5 μM of simvastatin stimulation. All the angiogenesis-related factors showed a slight inhibitory effect with the increasing drug concentration. Thus, we demonstrated that simvastatin could promote not only osteogenesis but also angiogenesis by regulating macrophage polarization.

## 4. Discussion

In this study, we found the inhibitory effect of simvastatin on M1 macrophages and demonstrated the ameliorative effect of simvastatin on MC3T3-E1 osteogenic differentiation as well as HUVECs’ angiogenesis. Interestingly, the effect of simvastatin on osteogenic differentiation and vascularization occurred through the modulation of macrophage polarization. Thus, simvastatin not only directly promotes vascular regeneration and bone regeneration, but the immune-related microenvironment stimulated by simvastatin indirectly also affects the functional behavior of MC3T3-E1 and HUVECs, favoring bone regeneration.

Substantial evidence suggests that vascular endothelial cells and preosteoblasts play a crucial role in the regulation of osteogenesis in the skeletal system [37]. Previous findings have confirmed that simvastatin acting on HUVECs and MC3T3-E1 can effectively promote cell differentiation, which is conducive to bone and blood vessel formation [31,38]. In this study, we confirmed that simvastatin could effectively promote the differentiation and osteogenesis of MC3T3-E1 cells. Simvastatin at a concentration of 2 μM significantly increased ALP activity and the expression of ALP and RUNX2 and promoted calcium deposition (Figure 1). It is well known that simvastatin can effectively promote the expression of osteogenic factors ALP and RUNX2, increase mineralized nodule formation, and enhance the activity of ALP on mesenchymal stem cells [39]. During osteogenesis, Runx2 is a very important upstream transcription factor in osteoblast differentiation, and ALP is one of the indicators that directly reflects the degree of osteoblast differentiation and evaluates the ability of osteoblast differentiation and mineralization [40]. When simvastatin acts on preosteoblast MC3T3-E1, it can promote cell differentiation and facilitate mineralization deposition by enhancing alkaline phosphatase (ALP) activity, which is positively correlated with dose and time [41]. These findings suggest that simvastatin effectively acts on MC3T3-E1 to induce osteogenic differentiation, consistent with our results. Furthermore, the pro-angiogenic activity of simvastatin was assessed using cell migration and tube formation, and the results showed that simvastatin enhanced the migration and tube formation of HUVECs’ immunofluorescence and RT–qPCR results showed that simvastatin remarkably enhanced the fluorescence intensity and the gene expression of CD31, VEGF, and HIF-1α, indicating that simvastatin stimulated the angiogenic differentiation of HUVECs (Figure 2 and Figure 3). VEGF is an essential pro-angiogenic cytokine, which exerts its functions mainly via binding to VEGFR2 on the membrane of endothelial cells (ECs) [42]. During bone development, angiogenesis and osteogenesis are physiologically coupled through VEGF expression, which can promote the migration and proliferation of endothelial cells and indirectly stimulate osteogenesis [43]. Simvastatin has been reported to promote wound healing by increasing VEGF expression in experimental diabetes [44]. Hypoxia-inducible factor (HIF-1) is a key transcriptional regulator of the cellular response to hypoxia and consists of HIF-1α and HIF-1β subunits, of which VEGF is the main target gene of HIF-1α [45]. Evidence suggests that the HIF-1α/VEGF pathway is involved in osteogenesis and angiogenesis [43,46]. Our previous studies showed that the Sim@PLGA/Gel-CS bone regeneration membrane effectively promoted angiogenesis and bone regeneration [47]. These findings suggest that simvastatin at appropriate concentrations effectively acts on the MC3T3-E1 and HUVECs to induce osteogenesis and angiogenesis. In addition, we found that simvastatin could significantly promote osteogenic differentiation at a concentration of 2 μM, and it could substantially promote angiogenesis at concentrations of 0.1 μM and 0.5 μM.

Following bone tissue injury, immune responses are rapidly triggered and play an essential regulatory role in initiating and coordinating subsequent osteogenesis and angiogenesis during tissue repair. [48]. Macrophages, as key innate immune effector cells, play a key role in regulating the immune response of the host, can be polarized into different phenotypes, including pro-inflammatory M1-type and pro-healing M2-type, and the macrophage phenotype depends on the stage of the repair process and the specific tissue microenvironment [49]. M2-type macrophages are often considered to be closely related to tissue repair, inducing bone formation and secreting osteogenic cytokines such as BMP2 and VEGF during osteogenesis [50]. In bone tissue-engineered immunomodulation studies, timely tissue repair is related to the transformation from pro-inflammatory M1 macrophage to the anti-inflammatory M2 phenotype [51]. Chow SK et al. [10] reported that an M1 macrophage-induced increase in pro-inflammatory cytokines initiated a series of anti-inflammatory signals that subsequently guided the transition to an M2-dominated anti-inflammatory phase [10]. To further explore the potential effects of simvastatin on the phenotypic transformation of macrophages, we focused on simvastatin-induced changes in M1 macrophages as it is essential for the initiation of the regeneration process. In this experiment, we found that simvastatin at an appropriate concentration could effectively inhibit the generation of M1 macrophages (Figure 4). Therefore, we hypothesized that simvastatin inhibits M1 macrophage polarization, which may be conducive to tissue repair. We also found that BMP2 and VEGF expression levels in macrophages increased in a dose-dependent manner with rising concentrations of simvastatin (Figure 5A,B). Therefore, we hypothesized that the modulatory effect of simvastatin on macrophage phenotype might further affect endothelial cells and osteoblasts, and we further confirmed this hypothesis by transwell co-culture experiments. 

With the understanding of bone immunity, macrophages are important regulatory cells for bone regeneration and vascularization [52]. Macrophages secrete anti-inflammatory cytokines such as IL-10 and express enzymes like Arg-1, as well as pro-inflammatory cytokines such as IL-6 and TNF-α, which can affect bone formation [53]. Stefanowski, J. et al. [54] found that macrophages were closely related to H-type endothelial cells during the regenerative events of fracture healing and were actively involved in the initial reconstruction of the vascular network, which is a key aspect of their role in the healing of bone defects [54]. Therefore, in addition to directly regulating vascular endothelial cells and preosteoblasts, regulating macrophages can be used as an indirect therapeutic strategy to promote bone regeneration and angiogenesis. However, the effect of simvastatin on bone regeneration and angiogenesis through macrophage regulation had not been studied. Therefore, the influence of the immune microenvironment generated by macrophages upon simvastatin stimulation on the functions of MC3T3-E1 and HUVECs was investigated in this study. 

MC3T3-E1 cells and HUVECs were cultured in a transwell coculture system with simvastatin-treated macrophages separately. We found mineralized particles’ formation and osteogenesis-related genes, including the Runx2 and ALP secretion of MC3T3-E1 cells, were upregulated after incubation with the simvastatin-treated macrophages (Figure 5C,D). It could be seen that simvastatin promoted MC3T3-E1 osteogenic differentiation by the immunoregulation of macrophages, and the optimal concentration of simvastatin in promoting osteogenesis was 1 μM. Meanwhile, under co-culture conditions, angiogenic ability, osteogenesis-related gene, and immunofluorescence intensity expression (CD31, VEGF, and HIF-1α) were enhanced at an appropriate concentration (Figure 6). Our results showed that simvastatin enhanced the angiogenic effect of macrophages, and the concentrations of simvastatin promoting angiogenesis were 0.1 μM and 0.5 μM. These results indicate simvastatin can alter the immunomodulatory functions of macrophages to promote osteogenesis and angiogenesis. Interestingly, we found simvastatin could promote osteogenesis and angiogenesis through macrophage regulation at a lower concentration than simvastatin alone, suggested that simvastatin-treated macrophages may have a more pronounced effect on promoting angiogenesis in HUVECs and bone regeneration in MC3T3-E1 cells compared to the direct effects of simvastatin on these cells. We therefore hypothesize that, in addition to the increased secretion of growth factors induced by simvastatin, the reduction in inflammatory cytokine secretion by macrophages also contributes to enhanced osteogenesis and angiogenesis, thereby facilitating bone tissue repair. In addition, the promotion of M2 macrophages by SIM may also play a role here, which we will further verify in future experiments [55].

## 5. Conclusions

This study confirmed that simvastatin could inhibit the polarization of macrophages into the M1 phenotype and promote the expression of bone regeneration-related factors. Meanwhile, simvastatin improved the osteogenic differentiation of MC3T3-E1 and promoted the angiogenesis potential of HUVECs. In addition, the immunoregulation of simvastatin on macrophages contributed to the osteogenesis of MC3T3-E1 and angiogenesis of HUVECs. Therefore, we consider simvastatin to be a promising drug for promoting bone regeneration, not only because of its pro-regenerative properties, but also because it plays an important role in bone healing by modulating the immunomodulatory microenvironment.

## Figures and Tables

**Table 1:** Primer sequences for RT–qPCR analyses.

Gene	Forward Primer (5’-3’)	Reverse Primer (3’-5’)
Mu GAPDH	AGGAGCGAGACCCCACTAACA	AGGGGGGCTAAGCAGTTGGT
Mu CD86	CTGCTCATCATTGTATGTCAC	ACTGCCTTCACTCTGCATTTG
Mu BMP2	AACGAGAAAAGCGTCAAGCC	AGGTGCCACGATCCAGTCAT
Mu VEGF	AGGAGTACCCCGACGAGATAGA	CACATCTGCTGTGCTGTAGGAA
Mu RUNX2	AGCGGACGAGGCAAGAGTTT	AGGCGGGACACCTACTCTCATA
Mu ALP	TGAATCGGAACAACCTGACTGA	GAGCCTGCTTGGCCTTACC
Hu β-Actin	TGGCACCCAGCACAATGAA	CTAAGTCATAGTCCGCCTAGAAGCA
Hu VEGF	CTGCTCTACCTCCACCATGC	GGAAGATGTCCACCAGGGTC
Hu CD31	CCAGGCCAGCAGTACCACTT	ACGTCTGAGTTCAGAGGCTCTTT
Hu HIF-1α	GGACAGTACAGGATGCTTGCC	TGCTGAATAATACCACTCACAACG

## Data Availability

Data presented in this study are available on request from the corresponding author.

## Abbreviations

ALP	Alkaline Phosphatase
BMP2	Bone Morphogenetic Protein-2
CCK8	Cell Counting Kit-8
CD31	Cluster of Differentiation 31
CD86	Cluster of Differentiation 86
cDNA	Complementary DNA
DAPI	4′,6-Diamidino-2-Phenylindole
DMEM	Dulbecco’s Modified Eagle Medium
ECM	Endothelial Cell Medium
FBS	Fetal Bovine Serum
GAPDH	Glyceraldehyde-3-Phosphate Dehydrogenase
HIF-1α	Hypoxia-Inducible Factor-1 Alpha
HMG-CoA	Hydroxymethylglutaryl-Coenzyme A
HUVECs	Human Umbilical Vein Endothelial Cells
IFN-γ	Interferon-gamma
IL	Interleukin
iNOS	Inducible Nitric Oxide Synthase
LPS	Lipopolysaccharide
MC3T3-E1	Mouse Calvaria-derived Pre-osteoblastic Cell Line
M1	Classically Activated Macrophage (Pro-inflammatory)
NO	Nitric Oxide
PBS	Phosphate Buffered Saline
PS	Penicillin-Streptomycin
ROS	Reactive Oxygen Species
SYBR	SYBR Green Nucleic Acid Stain
TNF-α	Tumor Necrosis Factor-alpha
VEGF	Vascular Endothelial Growth Factor
